# Serum Uric Acid and Left Ventricular Mass in Essential Hypertension

**DOI:** 10.3389/fcvm.2020.570000

**Published:** 2020-11-26

**Authors:** Valeria Visco, Antonietta Valeria Pascale, Nicola Virtuoso, Felice Mongiello, Federico Cinque, Renato Gioia, Rosa Finelli, Pietro Mazzeo, Maria Virginia Manzi, Carmine Morisco, Francesco Rozza, Raffaele Izzo, Federica Cerasuolo, Michele Ciccarelli, Guido Iaccarino

**Affiliations:** ^1^Department of Medicine, Surgery, and Dentistry, University of Salerno, Salerno, Italy; ^2^Cardiology Unit, University Hospital San Giovanni di Dio e Ruggi d'Aragona, Salerno, Italy; ^3^Cardiology Unit, Maria SS. Addolorata Hospital, Salerno, Italy; ^4^Department of Medical and Surgical Sciences, University of Foggia, Foggia, Italy; ^5^Department of Advanced Biomedical Sciences, Federico II University of Naples, Naples, Italy; ^6^Interdepartmental Center of Research on High Blood Pressure and Related Conditions “CIRIAPA”, Federico II University, Naples, Italy

**Keywords:** hypertension, uric acid, left ventricular mass, risk factors, hypertrophy

## Abstract

Serum uric acid (sUA) has been associated with cardiovascular risk. Although the recent mechanistic hypothesis poses the basis for the association between sUA and left ventricular mass index (LVMi), the issue remains poorly investigated in a clinical setup. Through a retrospective analysis of the database of the departmental Hypertension Clinic of University Hospital of Salerno Medical School, we identified 177 essential hypertensives (age 60.3 ± 13.3 years; 85 men), free from uric acid-modulating medications and severe chronic kidney disease, and whose sUA values, anthropometric, clinical, and echocardiographic data were available. In the studied cohort, the average duration of hypertension was 8.4 ± 7.1 years. LVMi associated with classical determinants, such as age, blood pressure, and kidney function, although after multivariate correction, only age remained significant. Also, sUA correlated positively with LVMi, as well as body size, metabolism, and kidney function. In a multivariate analysis, sUA confirmed the independent association with LVMi. Also, levels of sUA >5.6 mg/dl are associated with larger cardiac size. We confirmed our data in a replicate analysis performed in a larger population (1,379 hypertensives) from an independent clinic. Our results demonstrate that sUA increases with LVMi, and a cutoff of 5.6 mg/dl predict larger LV sizes. Our data suggest that hyperuricemia might help to stratify the risk of larger cardiac size in hypertensives.

## Introduction

Serum uric acid (sUA) is the final product of purine catabolism; its levels depend on the equilibrium of production and elimination, which occurs through the kidney. By routine, the normal distribution of sUA among the general population is considered below 6 mg/dl in women and 7 mg/dl in men (1). Hyperuricemia results from either an overproduction and/or a reduced UA renal excretion, thus explaining a complex interaction of physiological conditions and exogenous factors that can affect sUA (2).

In recent years, sUA has become an important parameter to consider when assessing cardiovascular risk. Indeed, sUA is associated with insulin resistance, hypertension, diabetes mellitus, dyslipidemia, obesity, metabolic syndrome, renal dysfunction, and hypothyroidism (3–5), which may explain enhanced cardiovascular (CV) risk among hyperuricemic individuals (6, 7). Noteworthy, ESC /ESH guidelines (8) have drawn new attention in the definition of CV risk based on sUA, capitalizing on a series of large studies reviewed by Borghi et al. (9); indeed, the 2018 European guidelines on the management of hypertension include sUA in the routine workup (8).

Recently, Kuwabara et al. showed that in the Japanese population, having higher sUA could be a risk for hypertension, dyslipidemia, and chronic kidney disease (CKD) (10).

Moreover, we reported that the optimal cut point for sUA to discriminate cardiovascular disease (CVD) mortality was 5.6 mg/dl (11) in population with high CV risk; in keeping with these results, the information on levels of sUA above or below 5.6 mg/dl incrementally predicted CVD mortality over Heart Score (11). The ability of sUA to predict CV risk probably associates with its ability to identify target organ damage (TOD), which participates in the definition of CV risk. For translational properties, it is possible to speculate that sUA and TOD indeed associate, as demonstrated, for instance, for renal dysfunction and atherosclerosis (3–5). Less evidence is available for the association of sUA and cardiac damage, in particular with increased left ventricle mass indexes (LVMi) (7, 12, 13). This is an independent, powerful predictor of CV morbidity and mortality in patients with hypertension and is associated with an increased incidence of arrhythmia, myocardial infarction, and stroke (14). Effective treatment can lead to regression of LVMi, in particular in young hypertensives, with a recent history of the disease, since superimposed age-related biochemical and histologic changes compromise LVMi response to therapy (15).

Our study aims to investigate the relationship between sUA and cardiac structural and functional variables in a cohort of hypertensive patients.

## Experimental Section

### Recording and Organizing Data

The population enrolled in the departmental Hypertension Clinic of University Hospital of Salerno Medical School is included in a central electronic database (Wincare, Gesan, Napoli, Italy), which contains separate electronic sheets for medical history, physical examination, clinical data, laboratory tests, electrocardiogram, and cardiac and vascular ultrasounds.

### Study Population

The database was interrogated to extract data of patients according to the following inclusion criteria: patients visited consecutively over a period of 3 months, with at least an average follow-up of 3 years, both males and females, age 18–80, with blood pressure <140/90 mmHg in office, on active antihypertensive treatment. We excluded those patients that were actively treated with diuretics (also including the 6 months before enrollment and during the follow-up), with sUA lowering agents, pregnant women, patients diagnosed with gout, malignancies, rheumatic disease, in chronic treatment with anti-inflammatory or anti-pain agents, and no use of daily doses of alcohol>3 drinks per day. Patients with chronic kidney disease at stage 4 and higher were also excluded. The diagnosis of hypertension was based according to current guidelines (8). Data were then checked for quality to exclude patients with incomplete databases. For a list of the independent variables considered in this cohort, please see Table 1.

**Table 1:** Clinical, serum biochemistry, and cardiac ultrasound parameters.

	**Overall (*n* = 177)**	**sUA>5.6 mg/dl (*n* = 51)**	**sUA ≤5.6 mg/dl (*n* = 126)**	***p***
**Clinical**				
Age, years	60.3, 13.3	60.0, 13.3	60.5, 13.3	0.71
Men, *n* (%)	85 (48.0)	33 (64.7)	52 (41.3)	**0.005**
BMI, kg/m^2^	28.9, 5.6	29.1, 5.3	28.8, 5.8	0.15
HR, beats/min	71.5, 12.3	72.1, 10.1	71.7, 13.2	0.51
SBP, mmHg	144.7, 19.2	146.7, 19.9	143.9, 18.9	0.74
DBP, mmHg	86.3, 12.1	87.1, 13.5	85.9, 11.5	0.64
Pulse pressure, mmHg	58.4, 14.1	59.6, 14.8	57.0, 13.8	0.96
ABI	1.3, 0.2	1.2, 0.2	1.3, 0.1	**0.01**
Duration of hypertension, years	8.4, 7.1	9.4, 7.8	8.0, 6.8	0.21
Current-smokers, *n* (%)	46 (27.5)	16 (32.0)	30 (25.6)	0.40
**Serum biochemistry**				
TC, mg/dl	193.7, 40.1	192.8, 41.2	194.0, 39.8	0.85
HDL, mg/dl	54.3, 14.9	48.4, 12.4	56.7, 15.2	**0.001**
LDL, mg/dl	114.5, 36.6	113.9, 39.7	114.7, 35.4	0.89
Triglycerides, mg/dl	124.6, 59.6	152.6, 61.2	113.3, 55.3	**0.001**
sGlucose, mg/dl	103.9, 22.00	110.9, 27.6	101.1, 20.3	**0.01**
sCreatinine, mg/dl	0.9, 0.2	1.0, 0.2	0.9, 0.2	**0.01**
sUrea, mg/dl	40.3, 11.5	43.0, 13.4	39.2, 10.5	**0.045**
eGFR, ml/min/1.73m^2^	91.9, 33.4	88.3, 36.06	93.4, 32.3	0.36
sUA, mg/dl	4.99, 1.30	6.52, 0.77	4.38, 0.88	**0.001**
**Cardiac ultrasound**				
LVMi, g/m^2.7^	51.4, 13.8	54.7, 15.2	49.1, 13.0	**0.04**
LVEF, %	65.9, 10.1	65.8, 11.1	66.9, 9.8	0.49
RWT, %	0.4, 0.1	0.4, 0.1	0.4, 0.1	0.57
LAD, mm	39.1, 5.8	39.0, 4.8	38.9, 6.2	0.56
E/A	0.9, 0.3	0.9, 0.3	0.9, 0.3	0.96
DT, ms	204.2, 56.0	207.5, 55.0	202.4, 56.5	0.60
E/E'	7.4, 2.1	6.9, 2.2	7.8, 2.0	0.15
IVSd, mm	11.4, 2.1	12.3, 2.3	11.0, 2.0	**0.001**

*BMI, body mass index; HR, heart rate; SBP, systolic blood pressure; DBP, diastolic blood pressure; ABI, ankle-brachial index; TC, total cholesterol; HDL, high-density lipoprotein; LDL, low-density lipoprotein; eGFR, estimated glomerular filtration rate; LVMi, left ventricular mass index; LVEF, left ventricular ejection fraction; RWT, relative wall thickness; LAD, left atrial diameter; E, early-wave transmitral diastolic velocity; A, late-wave transmitral diastolic velocity; E', early-diastolic velocity at tissue Doppler imaging; DT, deceleration time; IVSd, diastolic intraventricular septum diameter; sGlucose, serum glucose; sCreatinine, serum creatinine; sUrea, serum urea; sUA, serum uric acid. Data are presented as means ± SD unless otherwise indicated. p-values were calculated by unpaired t-test for continuous variables, or χ^2^ test for frequencies. Bold values indicate statistical significance (p < 0.05)*.

To replicate and validate the results of our study, we obtained the database of 1,379 hypertensive patients from the Hypertension Clinic of the Federico II University, which is located in the nearby city of Naples, in Campania, Italy. The Hypertension Clinic of the Federico II University uses a similar electronic chart for the management of outpatients (Wincare, Gesan), which has been in use for almost 40 years (16). Patients were selected from a database of over 25,000 hypertensives, according to the very same inclusion criteria described above.

Informed consent was obtained from each patient, and the study protocol conforms to the ethical guidelines of the 1975 Declaration of Helsinki as reflected in *a priori* approval by the institutions' human research committee (NCT03305276). The validation group data were obtained from the anonymized database of a previously approved, completed trial (ClincalTrials.gov: NCT00408512).

### Baseline Clinical Characteristics

The visit includes an update of anamnestic data and medical history with particular attention to lifestyle habits and pharmacological treatments, physical examination (including anthropometric measurements, blood pressure values, and heart rate), and standard electrocardiography.

Blood pressure (BP) measurement was obtained with an automated oscillometer (Afib screen, Microlife, Italy), validated according to international standardized protocols, and adequately maintained to verify calibration. Cuff placement was preceded by the selection of the appropriate cuff size for the patient's arm circumference; the lower end of the cuff was 2 to 3 cm above the antecubital fossa. The BP reading was obtained in the supine position (after a resting period of at least 10 min), in sitting and standing positions; the cuff was at the heart level, whatever the position of the patient. At least two BP measurements were recorded in every position (timed 1–2 min apart), and additional measurements were obtained if the first two assessments were largely different. In this study, we considered the average BP in the sitting position according to the current guidelines (8). In subjects resting in the supine position for 10 min, the ankle to brachial index (ABI) was determined using an automated oscillometric BP device at the right limb.

### Laboratory Analysis

Venous blood samples were collected in the morning after an overnight fast as a general rule. Blood chemistry was evaluated according to standardized methods by trained personnel. sUA levels were determined using the uricase-peroxidase system. Triglycerides and total and HDL cholesterol were assayed enzymatically, while LDL cholesterol was calculated with the formula of Friedewald. Serum glucose was measured using the glucose oxidase method. The estimated glomerular filtration rate (eGFR) was calculated by the Modification of Diet in Renal Disease (MDRD) equation.

### Cardiac Ultrasounds

All patients of this study received cardiac ultrasounds (Vivid E80, GE Healthcare) with M-mode, 2D, pulsed, and color-flow Doppler within 4 months from the first visit. To eliminate the confounding effect of gender on cardiac size, in this study, LVMi was adjusted for height ^2.7^ (8). Simpson's ejection fraction evaluated LV systolic function. A pulsed Doppler estimated the diastolic function from an apical four-chamber view at the level of mitral valve tips; early- (E) and late-wave (A) diastolic velocities and their ratio (E/A) were measured. Tissue Doppler imaging (TDI) was also performed, to calculate the E/E' ratio, where E′ is the early diastolic velocity at TDI measured at the septal and lateral corner of the mitral annulus.

### Statistic Analysis

The data are expressed as frequencies and percentages for qualitative variables and as mean ± standard deviation (SD) for quantitative ones. Using ANOVA, we analyzed continuous variables; categorical data were compared using the χ^2^ test. Linear univariate regression analyses, with confidence intervals, were tested on sUA and LVMi; multivariable regression analyses were performed on the significant continuous and categorical variables. Statistical analysis was performed using SPSS software for Windows, version 26.0 (SPSS Inc, Chicago, IL, United States).

## Results

### Population Features

We enrolled 300 hypertensives consecutively admitted to our clinic from February to May 2017; we followed up for 12 months to collect all clinical, cardiac US, and serum biochemistry data. After 1 year of follow-up, 31 patients were lost, and 92 were discarded for the incompleteness of the data. The analysis was therefore completed on 177 patients. The clinical, biochemical, and cardiac ultrasound (US) parameters of the study patients are summarized in Table 1. Men had greater LVMi and intraventricular septum dimensions than women (data not shown).

### Anthropometric and Hemodynamic Parameters Influencing LVMi

LVMi was significantly and directly correlated with age, serum glucose, blood pulse pressure, and kidney function (Table 2). Interestingly, LVMi was significantly and inversely related to HDL (Table 2). After a correction in a multivariate analysis, only age significantly correlated with LVMi (Table 3).

**Table 2:** Univariable linear regression analysis with LVMi.

	**Beta**	***p***	**Lower 95%**	**Upper 95%**
**Clinical**				
Age	0.337	**0.001**	0.199	0.492
Gender	0.083	0.27	−1.833	6.369
HR	−0.078	0.31	−0.253	0.080
SBP	0.157	**0.04**	0.008	0.220
DBP	−0.048	0.53	−0.214	0.126
Pulse pressure	0.255	**0.001**	0.103	0.387
ABI	0.099	0.22	−5.609	23.949
**Serum biochemistry**				
TC	−0.089	0.24	−0.081	0.021
HDL	−0.151	**0.046**	−0.277	−0.003
LDL	−0.031	0.68	−0.068	0.044
Triglycerides	−0.014	0.86	−0.038	0.031
sGlucose	0.201	**0.007**	0.033	0.208
sCreatinine	0.159	**0.04**	0.703	18.662
sUrea	0.179	**0.02**	0.038	0.39
eGFR	−0.109	0.15	−0.106	0.016
sUA	2.099	**0.009**	0.539	3.659

*SBP, systolic blood pressure; DBP, diastolic blood pressure; HR, heart rate; TC, total cholesterol; HDL, high-density lipoprotein; LDL, low-density lipoprotein; sGlucose, serum glucose; sCreatinine, serum creatinine; sUrea, serum urea; eGFR, estimated glomerular filtration rate; sUA, serum uric acid. Bold values indicate statistical significance (p < 0.05)*.

**Table 3:** Multivariate linear regression analysis with LVMi.

	**Beta**	***p***	**Lower confidence limit**	**Upper confidence limit**
Age	0.306	**<0.0001**	0.173	0.463
SBP	0.127	0.07	−0.008	0.19
HDL	−0.118	0.11	−0.244	0.026
sGlucose	0.138	0.052	−0.001	0.166
sCreatinine	0.04	0.64	−7.655	12.469
sUrea	0.12	0.14	−0.046	0.333

*SBP, systolic blood pressure; HDL, high-density lipoprotein; sGlucose, serum glucose; sCreatinine, serum creatinine; sUrea, serum urea. Bold values indicate statistical significance (p < 0.05)*.

### Association Between sUA and LVMi

**Figure 1:**
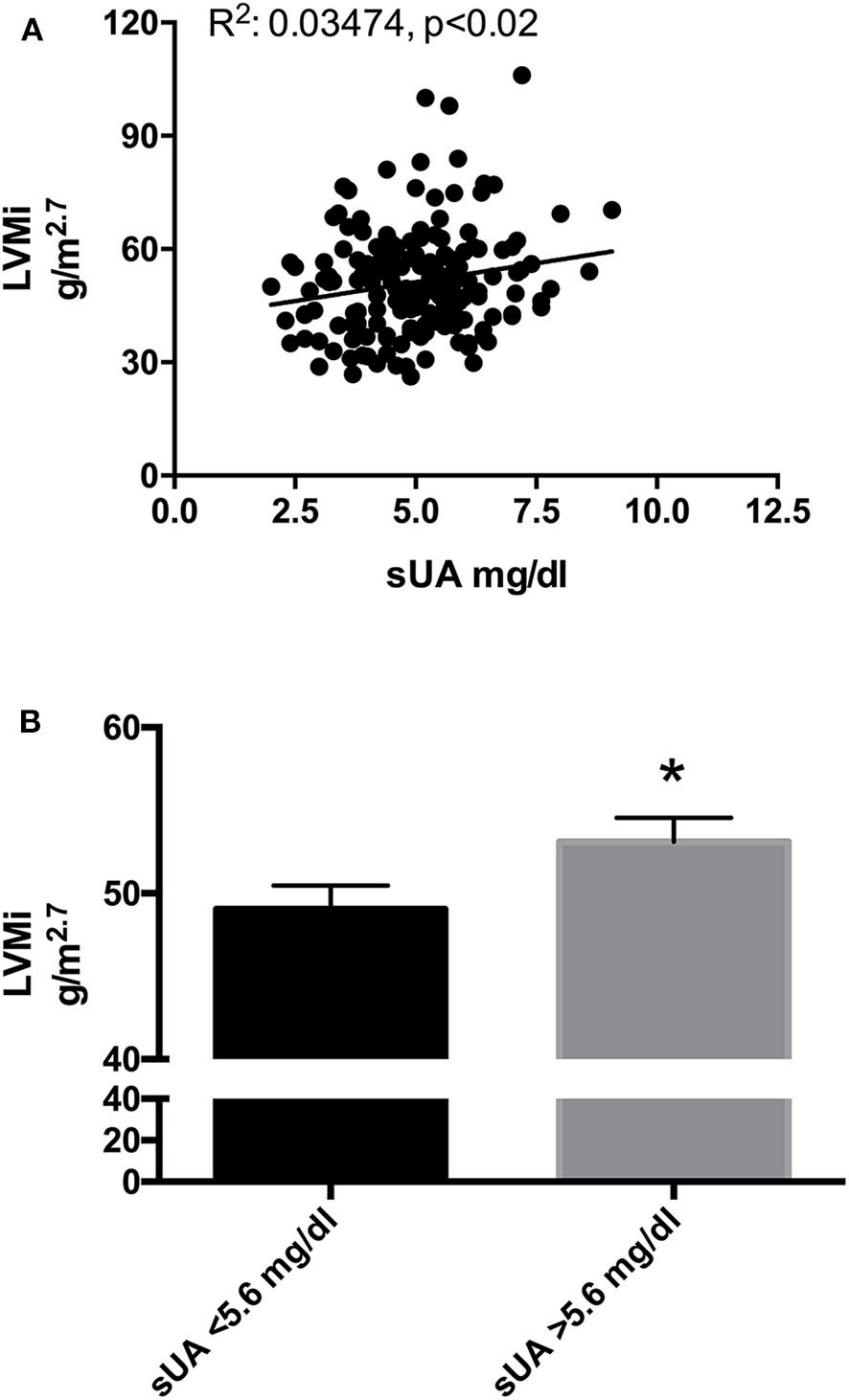
**(A)** Scatter plots of left ventricular mass index (LVMi) vs. serum uric acid (sUA) in the original population (177 patients). The graph shows that LVMi was significantly and directly related with sUA (*p* = 0.01). **(B)** LVMi in patients with sUA ≤ 5.6 mg/dl (left graph) and in patients with sUA >5.6 mg/dl (right graph). The graph shows higher value of LVMi in hypertensives with sUA > 5.6 mg/dl (*p* = 0.045). *Indicates *p* < 0.05.

To verify the association of sUA and cardiac TOD, we assessed the correlation between sUA on LV parameters. A statistically significant linear correlation was found between sUA and LVMi (Figure 1A); also, the univariate analysis indicated a positive relationship between the two parameters [Beta: 2.099; *p* < 0.009; (95% CI: 0.539–3.659)]. Furthermore, sUA levels displayed a significant correlation with gender, metabolism (BMI, weight, sGlucose, triglycerides, and HDL), and kidney function (creatinine, serum urea, eGFR) (Table 4).

**Table 4:** Univariate linear regression analysis with sUA.

	**Beta**	***p***	**Lower 95%**	**Upper 95%**
**Clinical**				
Age	0.012	0.87	−0.013	0.016
Gender	0.330	**0.001**	0.488	1.214
Weight	0.235	**0.001**	0.007	0.029
BMI	0.162	**0.03**	0.003	0.071
HR	0.007	0.93	−0.015	0.016
SBP	0.068	0.36	−0.005	0.015
DBP	0.083	0.30	−0.007	0.025
ABI	−0.096	0.23	−2.214	0.539
Pulse pressure	0.022	0.77	−0.012	0.016
Duration of hypertension	0.061	0.45	−0.016	0.038
**Serum biochemistry**				
TC	0.0047	0.36	−0.007	0.003
HDL	0.13	**<0.001**	−0.04	-0.016
LDL	0.0015	0.60	−0.007	0.004
Triglycerides	0.08	**<0.001**	0.003	0.009
sGlucose	0.03	**0.02**	0.002	0.018
sCreatinine	0.12	**<0.001**	1.182	2.779
sUrea	0.026	**0.03**	0.002	0.035
eGFR	0.026	**0.03**	−0.006	0.005
**Cardiac ultrasound**				
LVMi	0.186	**0.01**	0.005	0.032
LV ejecton fraction	0.0043	0.39	−0.028	0.011
RWT	<0.001	0.96	−2.032	2.505
LAD	0.015	0.12	−0.008	0.058
E/A	0.0005	0.78	−0.772	0.578
DT	0.006	0.36	−0.002	0.006
IVSd	0.02	0.09	0.035	0.212

*BMI, body mass index; SBP, systolic blood pressure; DBP, diastolic blood pressure; HR, heart rate; ABI, ankle-brachial index; TC, total cholesterol; HDL, high-density lipoprotein; LDL, low-density lipoprotein; sGlucose, serum glucose; sCreatinine, serum creatinine; sUrea, serum urea; eGFR, estimated glomerular filtration rate; LVMi, left ventricular mass index; RWT, relative wall thickness; LAD, left atrial diameter; E, early-wave transmitral diastolic velocity; A, late-wave transmitral diastolic velocity; DT, deceleration time; IVSd, diastolic intraventricular septum diameter. Bold values indicate statistical significance (p < 0.05)*.

Since previous literature had demonstrated that sUA might impact the outcome “death” on top of other determinants (11), also in this population, given the small number of patients, we tested the effect of sUA on LVMi in a multivariable analysis that included the above-indicated determinant, i.e., age and BMI. Both age and sUA independently correlate with LVMi (Table 5).

**Table 5:** Multivariate linear regression analysis of Age and sUA on LVMi.

**Model 1**	**Beta**	***p***	***Lower 95%***	***Upper 95%***
Age	0.32	**0.001**	0.2	0.465
BMI	0.374	**0.001**	0.598	1.228
sUA	0.133	**0.04**	0.04	2.786

*BMI, Body Mass Index; sUA, Serum Uric Acid. Bold values indicate statistical significance (p < 0.05)*.

Recently, we have proposed lower limits of sUA (5.6 mg/dl) to identify the increased risk of CV death (11). To verify whether this cutoff could also be helpful to identify a population with larger LVMi, we dichotomize our population by sUA > 5.6 mg/dl and observed a larger size of LV in hypertensives with sUA above the fixed threshold (Figure 1B).

### Replica Analysis in a Larger Population

To overcome some of the small sample size limitations of our study, we sought the collaboration of the Hypertension Outpatient Clinic of the Federico II University, which provided data regarding the first access to their database of 1,379 hypertensives selected out from a pool with over 25,000 patients, using the same inclusion criteria. The available clinical characteristics are illustrated in Table 6, grouped by sUA and gender.

**Table 6:** Clinical, serum biochemistry, and cardiac ultrasound parameters in the replica population.

	**Overall (*n* = 1,379)**	**sUA>5.6 mg/dl (*n* = 532)**	**sUA ≤5.6 mg/dl (*n* = 847)**	***p***	**Females (*n* = 587)**	**Males (*n* = 782)**	***p***
**Clinical**							
Age, years	52.9, 13.7	52.0, 14.6	53.5, 13.1	**<0.05**	55.9, 12.4	50.6, 14.1	**<0.001**
Men, *n* (%)	786 (57)	436 (82)	350 (42)	**<0.01**	587 (43)	782 (57)	**n.a**.
BMI, kg/m^2^	27.5, 4.4	28.6, 4.1	26.8, 4.4	**<0.01**	27.3, 4.9	27.5, 3.9	0.29
SBP, mmHg	143.7, 18.9	144.5, 18.5	143.2, 19.1	0.21	144.8, 19.8	142.2, 18.1	0.07
DBP, mmHg	88.9, 11.5	89.9, 11.6	88.3, 11.5	**<0.02**	87.8, 11.5	89.6, 11.4	**<0.006**
HR, beats/min	75.1, 27.9	74.2, 12.6	75.6, 34.2	0.39	74.9, 12.0	75.1, 35.2	0.86
**Serum biochemistry**							
Triglycerides, mg/dl	122.9, 69.4	138.9, 74.1	112.7, 64.1	**<0.01**	111.9, 58.0	130.9, 75.5	**<0.001**
TC, mg/dl	200.1, 38.1	200.8, 36.7	199.7, 38.9	0.60	206.0, 38.2	195.0, 37.2	**<0.001**
HDL, mg/dl	52.6, 13.7	48.6, 12.5	55.2, 13.9	**<0.01**	59.3, 13.5	47.7, 11.6	**<0.001**
LDL, mg/dl	122.6, 35.5	124.6, 33.3	121.3, 3, 696	0.12	125.7, 34.8	120.2, 35.8	**<0.034**
sGlucose, mg/dl	96.7, 20.5	98.4, 18.5	95.6, 21.6	**<0.01**	96.1, 24.3	97.0, 17.0	0.46
sCreatinine, mg/dl	0.9, 0.3	1.1, 0.3	0.9, 0.2	**<0.01**	0.8, 0.3	1.0, 0.2	**<0.001**
sUrea, mg/dl	37.4, 12.6	39.2, 13.5	36.3, 11.9	**<0.01**	37.7, 14.1	37.2, 11.3	0.47
sUA, mg/dl	5.2, 1.4	6.6, 0.9	4.3, 0.8	**<0.01**	4.4, 1.1	5.8, 1.3	**<0.001**
**Cardiac ultrasound**							
LVMi, g/m^2.7^	43.6, 9.0	44.2, 8.5	43.1, 9.2	**<0.05**	43.6, 9.4	43.5, 8.6	0.90
LVEF, %	67.5, 4.4	67.304.5	67.6, 4.4	0.30	67.8, 4.4	67.1, 4.4	**<0.01**
RWT, %	0.38, 0.04	0.38, 0.04	0.38, 0.04	0.98	0.38, 0.04	0.37, 0.03	**<0.013**
E/A	1.1, 0.3	1.1, 0.3	1.0, 0.3	0.10	1.0, 0.3	1.1, 0.3	**<0.001**

*BMI, body mass index; SBP, systolic blood pressure; DBP, diastolic blood pressure; HR, heart rate; TC, total cholesterol; HDL, high-density lipoprotein; LDL, low-density lipoprotein; sGlucose, serum glucose; sCreatinine, serum creatinine; sUrea, serum urea; sUA, serum uric acid. LVMi, left ventricular mass index; LVEF, left ventricular ejection fraction; RWT, relative wall thickness; E, early-wave transmitral diastolic velocity; A, late-wave transmitral diastolic velocity; Data are presented as means ± SE unless otherwise indicated. p-values were calculated by unpaired t-test for continuous variables, or χ^2^ test for frequencies. Bold values indicate statistical significance (p < 0.05)*.

**Figure 2:**
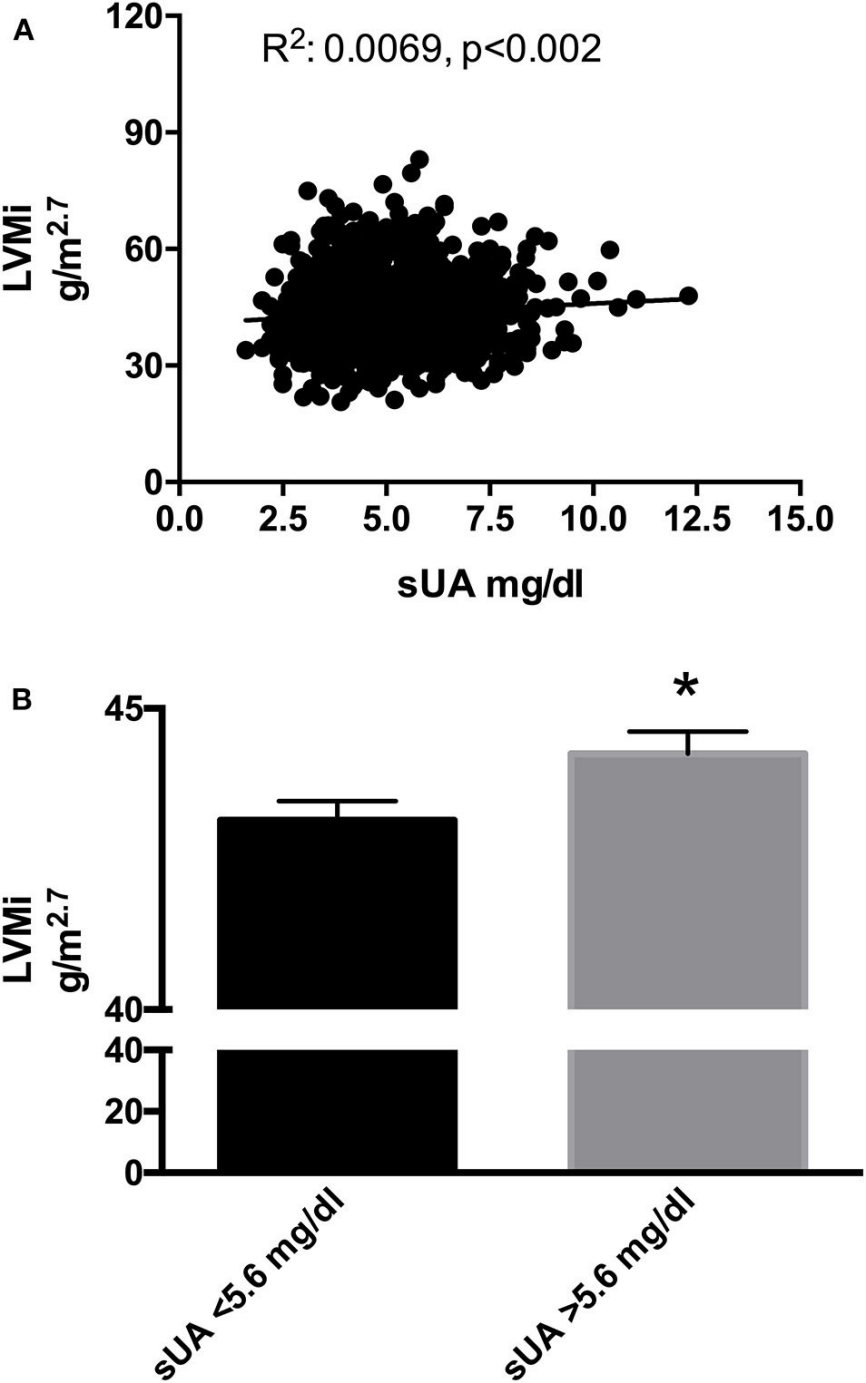
**(A)** Scatter plots of left ventricular mass index (LVMi) vs. serum uric acid (sUA) in the replica population (1,379 patients). The graph shows that LVMi was significantly and directly related with sUA (*p* = 0.01). **(B)** LVMi in patients with sUA ≤5.6 mg/dl (left graph) and in patients with sUA >5.6 mg/dl (right graph). The graph shows higher value of LVMi in hypertensives with sUA > 5.6 mg/dl (*p* = 0.02). *Indicates *p* < 0.05.

**Figure 3:**
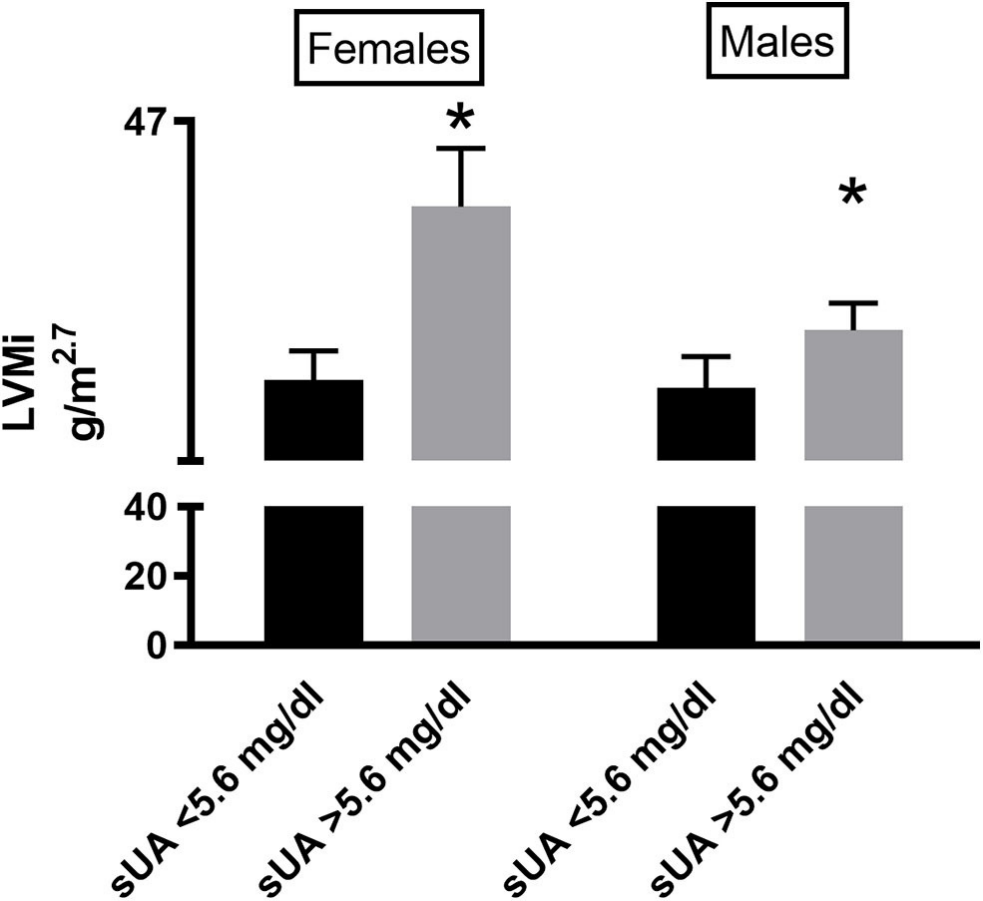
LVMi in female (left graph) and male (right graph) patients according to sUA ≤5.6 or sUA >5.6 mg/dl (right graph). The graph shows a higher value of LVMi in hypertensives with Sua >5.6 mg/dl (*p* < 0.05), independent from gender. *Indicates *p* < 0.05.

We then performed a correlation analysis between sUA and LVMi in this larger population, confirming the association between the two parameters, with a similar amplitude than that observed in the original smaller population (Figure 2A). In the univariable and multivariable analyses using the above-identified parameters, sUA remained significantly associated with LVMi (Table 7). Finally, when we dichotomize this population by the sUA cutoff of 5.6 mg/dl, we identify a population with a larger LVMi (Figure 2B). One might question the total independency of sUA with cardiac parameters based on the distribution of gender between sUA >5.6 and <5.6 groups. It is noteworthy that we used LVMi calculated by correction by height elevated at 2.7, a correction that reduces the differences of cardiac size indexes between genders. Anyway, we dichotomized the replica population according to gender. Figure 3 shows that sUA >5.6 identifies in both genders a population with larger LVMi.

**Table 7:** Univariate and multivariable linear regression analysis in the replica population.

	**Beta**	***p***	**Lower limit**	**Upper limit**
**Univariate analysis**				
**Clinical**				
Age	0.296	**<0.001**	0.160	0.226
Gender	−0.003	0.9	−1.019	0.895
HR	−0.080	**0.004**	−0.043	−0.008
SBP	0.161	**<0.001**	0.051	0.102
DBP	0.010	0.72	−0.034	0.050
**Serum biochemistry**				
TC	−0.005	0.85	−0.014	0.011
HDL	−0.085	**0.003**	−0.092	−0.019
LDL	−0.011	0.70	−0.018	0.012
Triglycerides	0.089	**0.001**	0.004	0.018
sGlucose	0.179	**<0.001**	0.055	0.102
sCreatinine	0.112	**<0.001**	1.895	5.413
sUrea	0.168	**<0.001**	0.080	0.158
sUA	0.068	**0.011**	0.097	0.765
**Multivariable analysis**				
Age	0.266	**<0.001**	0.140	0.209
SBP	0.140	**<0.001**	0.042	0.091
sGlucose	0.124	**<0.001**	0.032	0.078
sUA	0.070	**0.008**	0.116	0.772
Triglycerides	0.056	0.056	0.000	0.015
sCreatinine	0.040	0.22	−0.794	3.444
sUrea	0.052	0.11	−0.009	0.087
HR	−0.045	0.12	−0.030	0.004

*SBP, systolic blood pressure; DBP, diastolic blood pressure; HR, heart rate; TC, total cholesterol; HDL, high-density lipoprotein; LDL, low-density lipoprotein; sGlucose, serum glucose; sCreatinine, serum creatinine; sUrea, serum urea; sUA, serum uric acid. Bold values indicate statistical significance (p < 0.05)*.

## Discussion

Our results show for the first time in a real-life situation that sUA is in direct correlation with the LVMi, independently from other confounders. Furthermore, the use of the newly proposed cutoff of 5.6 mg/dl can help to predict the hypertensive population with a larger cardiac size. Our results agree with the growing role that this biochemical parameter plays in the definition of the CV risk of events in the hypertensive population.

Our data have been confirmed in two independent populations admitted at the University of Salerno and the Federico II University Hypertension Clinics. Apart from the confirmation of all the major observations, it is interesting to note that the association between sUA and LVMi comes with very low levels of *R*^2^, thus indicating that the two phenomena (sUA and LVMi), although associated, have distant mechanisms underlying. Nevertheless, the use of sUA in the management of hypertension might be helpful to identify patients with TOD.

The precise mechanism underlying the relationship between sUA and LVMi is still undetermined. It is possible to advocate pathophysiological mechanisms linking the two phenotypes. It has been reported that sUA increases tumor necrosis factor-alpha, stimulates mitogen-activated protein kinases, and activates the renin–angiotensin system, all of which are known to promote cardiac hypertrophy (17, 18). Alternatively, Cicero et al. (19) reported that sUA associates with increased pulse wave velocity (PWV) and augmentation index, the gold standard to estimate arterial stiffness in patients with hypertension, and to cause the increase in LV afterload, the major determinant of LV hypertrophy. Finally, sUA levels might reflect the degree of xanthine oxidase activity and resultant oxidative stress, which plays an essential role in the development of increased cardiac size (20). Furthermore, allopurinol, via inhibition of xanthine oxidases, could induce regression of LV mass in humans in a broad spectrum of diseases, including CKD, ischemic heart disease, and type 2 diabetes mellitus (21–23). Most likely, though, the identification of a common pathophysiological mechanism is still far to be identified, and at the moment, sUA represents a powerful biomarker that associates with cardiac TOD.

The need for biomarkers for the stratification of CV risk among hypertensives is a sensitive issue. Indeed, in the general definition of hypertension, we include multiple intermediate phenotypes: males and females, easy and difficult to treat, young and old, lean and obese and so on (24, 25). In the attempt to identify novel biomarkers that facilitate the identification of patients with a greater risk to develop CV events, sUA levels recently attained renewed emphasis (8). In particular, increased LVMi is reported in hypertensive patients (7, 26, 27). The combination of hyperuricemia combined with increased LVMi is an independent and powerful predictor for CV events, including myocardial infarction, angina pectoris, congestive heart failure, cerebral infarction, and transient cerebral ischemia (26).

The association between sUA and LVMi was previously shown in a different condition. In 540 patients with CKD, sUA directly correlated with LVMi (28); similarly, a significant and independent relationship between sUA and LVMi was observed also in renal transplant recipients after adjustment for potential confounding factors (12). At the opposite, in hypertension, conflicting data exist. While some authors report no independent association of sUA levels with LV size (29, 30), other studies showed a gender-related association between sUA and LVMi (31, 32). The reasons for these different observations can relate to methodological differences and heterogeneity of patient characteristics. Also, the use of higher cutoff to define hyperuricemia could have caused a bias in selecting a very high-risk population, where other conditions (i.e., CKD) might play a confounding effect. In our study, we use the recently proposed cutoff of sUA ≥5.6 mg/dl to define hyperuricemia (11). This cutoff allows the association of sUA and LVMi to emerge regardless of age and gender. Our results, therefore, further confirm that this cutoff can help to identify hypertensives with TOD, which in turn increases the risk of CV events.

Nakanishi et al. demonstrated that in a sample of the general population without overt cardiac disease, elevated sUA was independently associated with subclinical LV dysfunction, assessed as abnormal LV global longitudinal strain (GLS). Interestingly, the authors do not report the association between sUA and classical US parameters of LV function such as ejection fraction (33). Our results confirm therefore that sUA is not significantly related to LVEF (Table 4); furthermore, the two groups do not differ in diastolic function parameters (Table 1) and sUA is not significantly related to them (Table 4). Since we have not collected GLS data, we cannot confirm the existence of a relationship between sUA and GLS dysfunction.

Some studies have shown that sUA is related to markers of increased vascular damage, such as a low ABI (34) and higher sUA levels are associated with peripheral artery disease in the US general population (35). In our study, we found such association; accordingly, several studies reported that higher sUA was associated with a low ABI in women (36, 37), potentially related to estrogen effects (38–40).

A corollary in our study is the significant correlation of sUA with metabolic parameters such as BMI, weight, HDL, sGlucose, and TG (Table 4). It is notorious that sUA can be sensitive to diet, as well as other metabolic patterns. There is now a large body of evidence supporting a role for dietary changes in management of BP and metabolic disorders (41) and we have recently established that the length of food supply chain plays a key role in determining the risk of metabolic syndrome in a population adhering to the Mediterranean diet (42). Moreover, several observational studies and randomized control trials have demonstrated associations between dietary patterns and sUA levels (43, 44); specifically, a report from the Dietary Approaches to Stop Hypertension (DASH)–Sodium randomized trial demonstrated that consuming the DASH diet significantly reduced sUA compared to a typical American diet (45). The existence of a relationship between sUA levels and metabolism is extensively reported in the literature, in particular, a close relationship with BMI and waist circumference. In the PLAD study, patients with higher BMI had significantly higher uric acid levels (46). The Framingham Study demonstrated that subjects with higher levels of sUA had significantly higher BMI (47). This relationship is also confirmed by the evidence that the stable reduction of body weight is associated with the reduction of sUA, as demonstrated in intervention studies on the reduction of body weight obtained by bariatric surgery or with the use of anorectic drugs (48, 49).

Independently from BMI, metabolic parameters correlate with sUA. Hikita et al. investigated the relationship between sUA, body fat distribution, and metabolic syndrome, and showed a significant direct correlation with triglycerides, visceral fat, and the Homa index (50). According to these results, our data demonstrate the presence of a significant and linear correlation between sUA levels and some metabolic parameters (BMI, weight, triglycerides, and blood glucose) (Table 4). Moreover, in our results, sUA is significantly and inversely related to HDL (Table 4).

### Study Limitations

LVMi assessment by echocardiography could be considered a limitation due to lower sensitivity compared to more precise technological assessments. On the other hand, the cardiac US is more accessible for the larger part of hypertensive patients, making our results more relevant for daily practice. We followed up the patients for 12 months to collect all clinical, cardiac US, and serum biochemistry data, and this could be considered a limitation since the data were not collected at the same time; we calculated that from the first visit to the collection of the data elapsed an average time of 52 ±89 days for serum data and of 11 ±65 days to execute the cardiac US. We believe that this time is close enough to consider the collection of data to be contemporary.

## Conclusions

In summary, our results in a small population and their replication in a larger cohort demonstrate that sUA directly correlates with LVMi and that a cutoff of 5.6 mg/dl can identify patients with larger left ventricular mass. Our data suggest that hyperuricemia is an early marker of increased left ventricular mass that can be used to identify a hypertensive population with cardiac TOD.

## Data Availability Statement

The raw data supporting the conclusions of this article will be made available by the authors, without undue reservation.

## Ethics Statement

The studies involving human participants were reviewed and approved by Comitato Etico Campania sud. The patients/participants provided their written informed consent to participate in this study.

## Author Contributions

VV, MC, and GI: conceptualization, formal analysis, investigation, resources, writing – original draft, and writing – review & editing. VV, AP, NV, FM, FCi, RG, RF, PM, CM, MM, RI, FR, FCe, MC, and GI: data curation. All authors contributed to the article and approved the submitted version.

## Conflict of Interest

The authors declare that the research was conducted in the absence of any commercial or financial relationships that could be construed as a potential conflict of interest.
